# Editorial: Personalized immunotherapy for cancer

**DOI:** 10.3389/fonc.2023.1171907

**Published:** 2023-03-07

**Authors:** Anna Pasetto, Yong-Chen Lu

**Affiliations:** ^1^ Department of Oncology, Oslo University Hospital, Oslo, Norway; ^2^ Department of Laboratory Medicine, Division of Clinical Microbiology, ANA Futura, Karolinska Institutet, Stockholm, Sweden; ^3^ Department of Pathology, Winthrop P. Rockefeller Cancer Institute, University of Arkansas for Medical Sciences, Little Rock, AR, United States

**Keywords:** cancer immunotherapies, personalized medicine, adoptive cell immunotherapy, T cell, checkpoint blockade immunotherapy

Cancer is a complex and challenging disease to treat, and we are just beginning to appreciate its heterogeneity among and within patients. Personalized therapies may harness the specific characteristics of each tumor to obtain a better clinical outcome compared to traditional treatment approaches. Immunotherapy is one of the promising approaches for personalized treatment; Adoptive cell therapy and neoantigen vaccines are two examples on how the immune system can be used and trained to recognize specific antigens, resulting in tumor regressions. The goal of this Research Topic is to address current challenges and provide solutions to the main problems emerging in the development of personalized immunotherapies for cancers. In this Research Topic, several important aspects have been covered by outstanding research or review articles, which will be introduced below. We hope these articles will inspire scientists to conduct research the next frontiers of personalized immunotherapies.

## 1 Adoptive cell therapy using TIL, CAR or TCR

Adoptive cell therapy using *ex vivo* expanded TILs (tumor infiltrating lymphocytes) has become an effective treatment for patients with metastatic melanoma. However, one of the major technical difficulties is how to expand anti-tumor TILs selectively without expanding bystander T cells. Yunger et al. provided a potential solution by utilizing a synthetic immune niche of immobilized CCL21 and ICAM1 in order to optimize TIL expansion. This approach resulted in a TIL product with a higher expansion rate and lower expression of exhaustion markers, compared to traditional methods.

Cryopreservation is a critical step for the adoptive cell therapy, and it might potentially damage the quantity and quality of T cells during the manufacturing process. Brezinger-Dayan et al. conducted an important study to evaluate the impact of cryopreservation on CAR-T cell products. This study compared fresh versus cryopreserved CD19 CAR-T cells in terms of phenotypes and *in vitro* anti-tumor reactivities, and fresh CAR-T cells demonstrated stronger anti-tumor reactivities in general. However, the authors concluded that using frozen CAR-T cells remained a viable option, since the cryopreservation did not appear to affect the clinical responses in the trial.

A phase I/II clinical trial is an important milestone to evaluate the safety and potential efficacy of a newly developed therapy, for example, an adoptive cell therapy. Importantly, the detection of immune-related adverse events, such as cytokine release syndrome, is critical for immunotherapy clinical trials. Here, Maggadóttir et al. carefully described a clinical trial design, which involved in the transient expression of anti-hTERT TCR in autologous T cells, coupled with dose escalation. This clinical trial will be used to evaluate the safety and tolerability of this T cell product for the treatment of patients with metastatic non-small-cell lung cancer.

## 2 Checkpoint blockade immunotherapy in head-and-neck cancers and lung cancer

Only a small fraction of patients with head and neck squamous cell carcinomas (HNSCCs) respond to the checkpoint blockade immunotherapy. In a Review Article, Chen et al. discussed the tumor-intrinsic factors and host-intrinsic factors potentially involved in the immunotherapy for HNSCCs, such as the mutational landscape and the abundance of immune cells in tumors. These factors might contribute to the creation of a highly heterogenic tumor microenvironment, resulting in mixed responses to the immunotherapy observed in these patients.

It remains challenging to predict clinical responses for lung cancer patients following immunotherapy. PD-L1 expression has been identified as an important biomarker to predict responses. Here, Shi et al. investigated the correlation between PD-L1 expression and CT-based radiomic features in patients affected by early-stage lung adenocarcinomas. The authors proposed that radiomics could be an effective, non-invasive method to predict PD-L1 expression in this patient group.

## 3 Prediction of immune epitopes

It has been technically challenging to predict immunogenic MHC class II epitopes. Xu et al. described a new bioinformatics tool based on a convolutional neural network model (FIONA), which could accurately predict MHC class II epitope binding and immunogenicity. This tool can be used to predict CD4^+^ T cell immune responses against tumors, followed by personalized treatments, such as personalized neoantigen vaccines.

We would like to take the opportunity to thank all authors who have contributed their excellent manuscripts in this Research Topic. We also feel grateful for the professional and constructive feedbacks provided by the reviewers. Most importantly, we hope you enjoy reading these outstanding articles, which provide precious insights and innovative solutions in this exciting field.

## Author contributions

All authors listed have made a substantial, direct, and intellectual contribution to the work and approved it for publication. AP and YL contributed equally.

